# Hypermethylation of the miR-155 gene in the whole blood and decreased plasma level of miR-155 in rheumatoid arthritis

**DOI:** 10.1371/journal.pone.0233897

**Published:** 2020-06-02

**Authors:** Bogdan Kolarz, Marek Ciesla, Magdalena Dryglewska, Ann K. Rosenthal, Maria Majdan

**Affiliations:** 1 College of Medical Sciences, University of Rzeszow, Rzeszow, Poland; 2 Department of Rheumatology and Connective Tissue Disease, Medical University of Lublin, Lublin, Poland; 3 Division of Rheumatology, Department of Medicine, Medical College of Wisconsin, Wauwatosa, WI, United States of America; Universitat des Saarlandes, GERMANY

## Abstract

**Objectives:**

miR-155 plays a critical role in the inflammatory process and in diseases such as rheumatoid arthritis (RA). miR155 gene expression is regulated by its gene promoter region CpG island methylation. Previous studies have shown inconsistent changes in circulating levels of mir-155 in RA patients. The aims of our study were to evaluate miR-155 levels in plasma, to investigate its gene methylation level, and to correlate these levels with RA disease activity.

**Methods:**

One hundred and twenty-five patients with RA, and 30 age and sex-matched healthy controls (HC) were enrolled. Whole blood and plasma samples were collected and stored at -80°C until analysis. DAS28 score at the time of the blood draw was used to assess RA disease activity. The methylation status of miR-155 host gene was determined in whole blood by quantitative real-time methylation-specific PCR (qPCR). miR-155 expression levels were evaluated by quantitative reverse transcription PCR.

**Results:**

We found significantly lower circulating miR155 levels in RA patients compared to HC. Interestingly, the miR-155 gene methylation level was significantly higher in RA patients than in HC. miR-155 levels did not correlate with ACPA or RF positivity or disease activity.

**Conclusions:**

We show here higher miR-155 methylation in whole blood and lower plasma miR155 expression in RA patients in comparison to HC. The evaluation of miR-155 host gene methylation status or miR155 plasma level might be a potentially useful marker in RA determination.

## 1 Introduction

The recognition of epigenetic mechanisms introduced a new aspect to understanding the etiopathogenesis of RA. These mechanisms transmit signals from environmental factors to cell nuclei impacting gene expression [1]. Epigenetic mechanisms include DNA methylation, histone modifications such as methylation, acetylation, ubiquitination, phosphorylation, and a large variety of non-coding RNAs (ncRNA). ncRNA are divided into small non-coding RNA (sncRNA) and long non-coding RNA (lncRNA) [2]. The first are a wide group of nucleotide transcripts less than 200nt in length, and consist of micro-RNA (miRNA), small interfering RNA (siRNA), PIWI-interacting RNA (piRNA), small nucleolar RNA (snoRNA) and small nuclear RNA (snRNA). They play a fundamental role by regulating gene expression at the post-transcriptional level.

sncRNAs act by blocking or deleting the complementary mRNA strands before translation begins [3]. miRNAs account for most sncRNA in eukaryotic cells. miRNAs are single strand RNAs 21–23 nts in length. Each miR usually affects a number of mRNAs and the specific mRNA is regulated by a various miRs [4]. They affect a wide range of developmental and cellular processes such as apoptosis, cell differentiation and proliferation, cellular migration, cell fate determination, pluripotency, neural plasticity and play a role in neurodegenerative disorders, cancers, infectious, and autoimmune diseases [5, 6].

The miR155 gene is on chr21:25,573,980–25,574,044 on a forward strand with references to the assembly GRCh38/hg38. The gene has one transcript variant named MIR155-201 (pre-miR-155) which consist of 65bp. MIR155 gene is located inside its host gene (MIR155HG, also known as B-cell integration cluster, BIC). Both MIR155 and MIR155HG are regulated by the same promoter region. The transcript consists of 1600 bp and includes 4 exons with the following lengths: 106 bp, 133bp, 85bp and 1276 bp. MiR-155-3p is located in the last exon [2]. Upstream (190bp) to the transcription start site (TSS) of MIR155HG is a CpG-rich region which was previously studied in reference to methylation analysis [7–9]. MiR-155 is one of the first sncRNAs described in RA [10, 11].

MiR-155 is an important mediator of inflammation via stimulation of Th1 and Th17 cells [12]. Prior studies demonstrated that miR-155 deficient mice are resistant to collagen-induced arthritis (CIA) and that mice with B cell-specific over-expression of miR-155 produce higher levels of TNFα [13, 14]. It is suggested that the miR-155-PU1 pathway positively regulates the B-cell antibody production in RA [15]. The range of cytosolic or nuclear proteins affected by miR-155 and involved in RA is quite wide. It includes cytotoxic T-lymphocyte-associated protein (CTLA-4), activation-induced cytidine deaminase (AID), suppressor of cytokine signaling protein 1 (SOCS1), histone deacetylase 4 (HDAC4), I*κ*B kinase ε (IKKε), myeloid differentiation primary response gene 88 (MyD88), SH2 domain-containing inositol 5′-phosphatase 1 (SHIP1), TAK1-binding protein 2 (TAB 2), CCAAT/enhancer-binding protein *β* (C/EBP*β*), and Fas-associated protein with death domain (FADD). In the immune system, miR-155 affects dendritic cell function leading to over-expression of IL6, IL-12, IL-23, TNF-α, IL-1β, INF-α/β). It also affects macrophages stimulating SHIP1, TNF-α and G-CSF, and TH17 cells leading to IL-17 elevation. These changes represent only a portion of the regulatory mechanisms involving miR-155 that are involved in RA pathogenesis [16, 17].

In the literature there are no reports concerning miR-155 promoter region methylation in diseases of autoimmune origin. However, this epigenetic modulation may play an important role in inflammation though modulation of miR-155 expression.

Many studies suggest a poor correlation of circulating miRs levels and local tissue levels i.e. synovial fluid, serum or plasma [18]. The aim of our study was to evaluate circulating levels of miR-155 and miR-155 gene methylation levels in RA patients in comparison to HC, and to correlate levels with disease activity.

## 2 Material and methods

A total of 152 individuals, 122 with RA, 82,4% female, aged 52,2±12,3 years (mean±SD) and 30 controls, 76,7% female, aged 53,2±8,1 years were enrolled. RA patients recruited to the study included those consecutively seen at the Department of Rheumatology and Connective Tissue Diseases, Medical University of Lublin, Poland during March 2016 to April 2017 and can be considered representative of a larger RA population. RA diagnosis was made according to the 2010 ACR/EULAR or 1987 ACR criteria for classification of RA depending on time of diagnosis. Exclusion criteria included the presence of any infection or another severe illness during hospitalization. The healthy controls (HC) were a group of patients with no joint complaint or diagnosed as osteoarthritis, with no inflammatory rheumatic and musculoskeletal diseases. Written informed consent was obtained from every participant before entering the study. The study was approved by the Bioethics Board at the Medical University in Lublin, protocol number KE-0254/7/2016.

RA patients were divided into 4 groups based on DAS28 scoring. High disease activity was defined as a DAS28score >5,1; 27,2%), moderate activity (DAS28 >3,2–5,1; 36,8%), low activity (>2,6–3,2; 15,2%) and remission (≤2,6; 20,8%). Whole blood and plasma samples were collected from patients and stored at -80°C until analysis. The characteristics of the groups are summarized in Table 1.

**Table 1:** Characteristic of the groups. HC healthy control.

Characteristics	RA N = 122	HC N = 30	Antibodies tested for 122 RA			
			RA RF + (22U), n = 82	RA RF -, n = 40	RA ACPA +, n = 104	RA ACPA -, n = 18
Age; mean (SD)	52.2 (12.3)	53.2 (8.1)	53.9 (12.4)	48.7 (11.5)	53.3 (11.7)	45.1 (14.1)
Females; n (%)	103 (84.4)	23 (76.7)	69 (84.1)	34 (85)	88 (84.6)	15 (83.3)
Disease duration [years]; mean (SD)	11.52 (9.2)	n/a	12.5 (10)	10.9 (7.8)	12.2 (9.7)	9.5 (5)
Rheumatoid Factor positive; n (%),	82 (67.2)	none	82 (100)	0	78 (75)	4 (22.2)
ACPA positive; n (%)	104 (85.2)	none	78 (95.1)	26 (65)	104 (100)	0
ESR; mean (SD)	31.5 (24.5)	14.6 (9.2)	35 (26.3)	25.2 (19.5)	32.3 (25.4)	28.7 (21.3)
CRP [mg/l]; mean (SD)	14.1 (25.4)	1.4 (1.7)	15 (28.2)	11.9 (19.4)	13.2 (25.7)	19.2 (26.3)
VAS PGA; mean (SD)	33.6 (26.7)	n/a	34.4 (28)	32.6 (24.1)	33.5 (27)	35.2 (24.5)
VAS PhGA; mean (SD)	28.7 (24.4)	n/a	29.1 (25.3)	28.5 (22.5)	28.7 (24.6)	30.1 (23.5)
**Treatment:**		n/a				
*At least on Methotrexate*,* n(%)*	*88 (72*.*1)*		56 (68.3)	32 (80)	74 (71.2)	14 (77.8)
*At least on Biologics*, *n (%)*	*44 (36*.*1)*		24 (29.3)	20 (50)	38 (36.5)	6 (33.3)
*At least on Steroids*,* n (%)*	*72 (59)*		49 (59.8)	23 (57.5)	59 (56.7)	13 (72.2)
**Single drug therapy, n (%)**	43 (35.2)		30 (36.6)	13 (32.5)	38 (36.5)	5 (27.8)
Metotrexate, n (%)	22 (18)		14 (17.1)	8 (20)	20 (19.2)	2 (11.1)
Biologist, n (%)	4 (3.3)		2 (2.4)	2 (5)	4 (3.8)	0
Steroids, n (%)	17 (13.9)		14 (17.1)	3 (7.5)	14 (13.5)	3 (16.7)
**Double drug therapy, n(%)**	49 (40.2)		33 (40.2)	16 (40)	41 (39.4)	8 (44.4)
*At least Methotrexate+Steroids*, *n (%)*	51 (41.8)		33 (40.2)	18 (45)	41 (39.4)	10 (55.6)
*At least Methotrexate+Biologics*, *n (%)*	36 (29.5)		20 (24.4)	16 (40)	30 (28.8)	6 (33.3)
*At least Steroids +Biologics*, *n (%)*	25 (20.5)		13 (15.9)	12 (30)	21 (20.2)	4 (22.2)
Methotrexate+Steroids, n (%)	30 (24.6)		22 (26.8)	8 (20)	24 (23.1)	6 (33.3)
Methotrexate+Biologics, n (%)	15 (12.3)		9 (11)	6 (15)	13 (12.5)	2 (11.1)
Steroids+Biologics, n (%)	4 (3.3)		2 (2.4)	2 (5)	4 (3.8)	0
**Triple line therapy, n (%)**	21 (17.2)		11 (13.4)	10 (25)	17 (16.3)	4 (22.2)
**No therapy, n (%)**	9 (7.4)		8 (9.8)	1 (2.5)	8 (7.7)	1 (5.6)

### 2.1 DNA extraction

DNA was extracted from 200 μl of frozen white cell fraction according to the manufacturer’s protocol using the GeneMATRIX Quick Blood DNA Purification Kit (silica spin columns, Eurx, Poland). DNA was eluted in 100μl and stored at -80°C until analysis.

### 2.2 Methylation studies

One μg DNA was converted by sodium bisulphite using the EZ DNA Methylation Gold Kit (Zymo Research, USA) according to the manufacturer’s recommendation.

Quantitative real-time methylation-specific PCR (qMSP) was used to analyze the methylation status. The *MIR155* Host Gene was evaluated by the following set of primers: 5’-GGGTTAATTGGTGAGGTAAGGTG -3’ [sense] and 5’-CAACACAATCTCTAATCCCAAACA -3’ [antisense]. Primers were designed by MethPrimer Software, version 1.0 and were complementary to the unmethylated target sequence. We have chosen a similar promoter region to that used by Yim et al. [7]. However, our amplicon was located 411 bp upstream while still remaining in the CpG island.

To normalize input of DNA after bisulfide conversion, the promoter region free of CpG sites in the Beta-actin gene (*ACTB*) were amplified. The *ACTB* primers were the following sequences: 5’-GGTGGTGATGGAGGAGGTTTAG-3’ [sense] and 5’-CCCTTAAAAATTACAAAAACCACAACC-‘3 [antisense]. Primers were flanking a similar region to that described by Menigatti M. et al., however their sequences were manually redesigned [19].

The qMSP reaction contained for *MIR155* 200 nM for each primer, *ACTB* 600 nM, as well as 2μl bisulfide treated DNA. PCR was performed by Power SybrGreen (Life Technologies) on COBAZ z480 Real Time PCR System under the following thermal cycling conditions: 95°C for 10min.–polymerase activation step, followed by 40 cycles: 95°C for 10 seconds and annealing/extension step at 63°C for 1 min., followed by melting-curve step. The ability of primers to amplify specific sequences was evaluated by using fully methylated and unmethylated DNA controls (EpiTect PCR Control DNA Set, Qiagen, Germany).

QMSP efficiency, both for *ACTB* and *MIR155* was evaluated based on the Livak and Schmittgen method [20]. Relative ratios based on Pfaffl method were applied to evaluate a fold-change in the methylation level [21].

### 2.3 MicroRNA isolation

MicroRNA was isolated from 300 μl of frozen plasma according to the manufacturer’s protocol using the miRCURY RNA Isolation Kit–Biofluids (Exicon, Denmark). 1,5 μg carrier RNA from bacteriophage MS2 (Roche, Germany) was added to each sample in accordance with kit recommendation. MicroRNA was eluted in 50 μl of elution buffer and stored at -80°C until analysis.

### 2.4 Reverse transcription and miR expression study

Before reverse transcription microRNA was diluted 40 times and transcribed to cDNA according to the instruction using Universal cDNA Synthesis Kit II (Exicon, Denmark). PCR was performed in a total volume of 10 μl using: 1 μl of LNA primers set (Exicon), 5 μl Exilent SYBR Green Master Mix (Exicon) and 4 μl diluted cDNA. The reaction was performed on COBAZ z480 Real Time PCR System following the conditions mentioned in the manufacturer’s protocol. Based on Exicon’s recommendation, hsa-miR-425-5p (assay no. 204337) was chosen as a control gene. Hsa-miR-155-5p (assay no. 204308) was tested as a target gene.

qRT-PCR efficiency, both for *miR-425* and *miR-155* were evaluated based on the Livak and Schmittgen method [20]. Normalized relative ratio based on Pfaffl method was applied to evaluate a fold-change in the expression level [21].

### 2.5 Statistical analysis

Depending on the distribution, as assessed by Shapiro-Wilk W test, quantitative values were presented as median (interquartile range), mean± SD or numbers with percentages. The Whitney-Mann U-test was used to evaluate differences in methylation level between two groups (controls vs. RA patients). Kruskal-Wallis ANOVA and multiple comparison analysis post-hoc testing were used to evaluate the differences between controls and patients divided into groups based on DAS-28 scoring. A p-value < 0.05 was considered statistically significant. Analysis was performed with STATISTICA Version 13 (StatSoft Inc., USA).

## 3 Results

### 3.1 MiR-155 gene methylation

**Fig 1:**
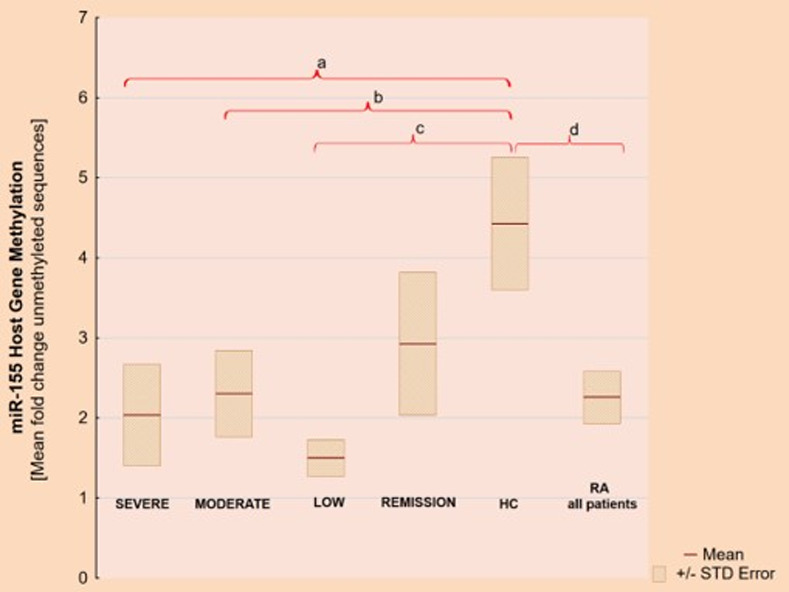
MiR-155 gene methylation level showed as unmethylated sequences–mean fold change. a) p = 0,0008, b) p = 0,0002, c) p = 0,01, d) p = 0,00001.

We found differences in miR-155 gene methylation levels between patients with RA and HC (p = 0,00001). Specifically, we found statistically significant higher miR155 methylation (lower unmethylated sequences fold-change) in: the high activity RA group vs HC (p = 0,0008), the moderate RA activity group vs HC (p = 0,0002) and the low RA activity group vs HC (p = 0,01). Results are presented in Fig 1.

### 3.2 MiR-155 plasma expression

**Fig 2:**
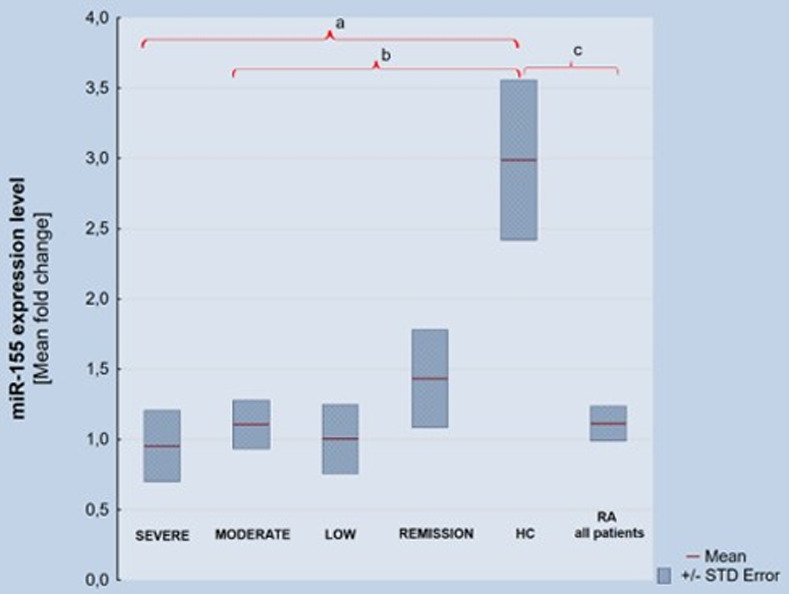
MiR-155 plasma expression level a) p = 0,0002, b) p = 0,009, c) p = 0,00004.

There were also differences in the plasma levels of miR-155 (p = 0,00004) in RA patients vs HC. miR-155 plasma expression was lower in the high RA activity group vs HC (p = 0,0001) and the moderate RA activity group vs HC (p = 0,009). In the RA group we did not find significant differences between various DAS28 RA activity subgroups. Results are shown in Fig 2. The results of miR-155 plasma concentration and its gene methylation level in RA according to DAS28 activity and in HCs are show in Table 2.

**Table 2:** Methylation level of miR-155 gene (shown as unmethylated sequences) and miR-155 plasma expression in RA group (divided according DAS28 activity) and in HC.

Group	miR-155 Methylation [unmethylated sequences]		miR155 Expession	
	Mean Fold-change	STD Error	Mean Fold-change	STD Error
RA High Activity DAS28 >5.1;n = 33; 27%	2,04	0,63	0,95	0,25
RA Moderate Activity DAS28 >3.2–5.1; n = 46; 37.7%	2,34	0,55	1,10	0,17
RA Low Activity DAS28>2.6–3.2; n = 17; 13.9%	1,50	0,23	1,0	0,25
RA Remission Activity DAS28≤2.6;n = 26; 21.3%	2,93	0,89	1,43	0,35
RA overalln = 122	2,24	0,32	1,11	0,12
HC n = 30	4,43	0,83	2,97	0,56

The raw data are attached as S1 Table.

### 3.3 Other results

**Fig 3:**
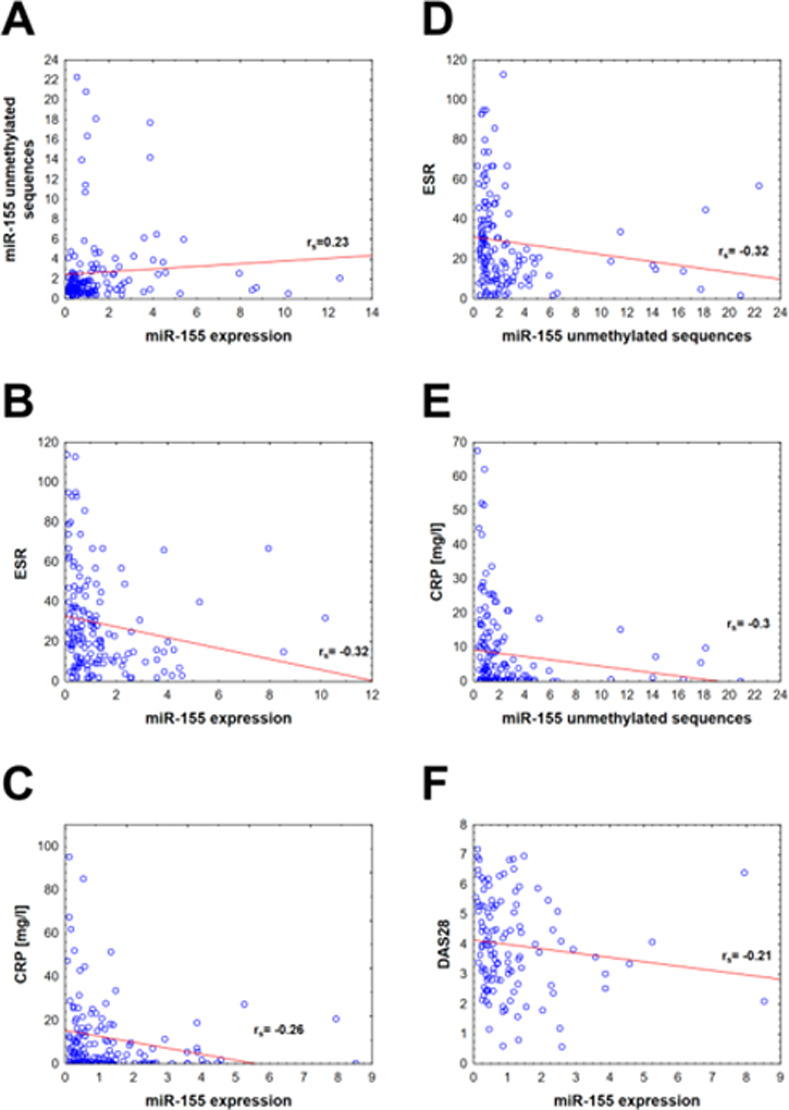
Spearman's rank correlation between studied parameters. All presented variables are statistically significant with the p-value <0.05. Diagrams from A to F show correlations between two variables and the following correlation coefficients (r_s_): **A:** correlation between miR-155 unmethylated sequences and miR-155 expression, r_s_ = 0,23; **B:** correlation between ESR and miR-155 expression, r_s_ = -0,32; **C:** correlation between CRP and miR-155 expression, r_s_ = -0,26; **D:** correlation between ESR and miR-155 unmethylated sequences, r_s_ = -0,32; **E:** correlation between CRP and miR-155 unmethylated sequences, r_s_ = -0,3; **F:** correlation between DAS28 and miR-155 expression, r_s_ = -0.21.

Furthermore, we found no statistical differences in miR-155 expression or its gene methylation between ACPA and RF positive or negative patients in the RA group. Results are shown in Table 3. Interestingly, there was a positive correlation between ESR and CRP and miR-155 methylation level and a negative correlation with miR-155 plasma expression. There was also a negative correlation between DAS28 and miR-155 plasma concentration. The results are presented in Fig 3. Our results data also show that there was a trend for methylation level of miR-155 to diminish and miR-155 plasma expression to increase in correlation with decreasing RA activity, but this was not statistically significant.

**Table 3:** Methylation level of miR-155 gene (shown as unmethylated sequences) and miR-155 plasma expression in RA patients according ACPA and RF positivity.

White cell fraction DNA miR-155 methylation level (unmethylated sequences)					Plasma miR-155 expression				
	Mean Fold-change	SD	STD Error	p-value		Mean Fold-change)	SD	STD Error	p-value
**ACPA + n = 104**	2.44	3.96	0.39	0.61	**ACPA + n = 104**	1.02	1.1	0.11	0.92
**ACPA—n = 18**	1.37	0.85	0.21		**ACPA n = 18**	1.8	2.38	0.61	
**RF+ n = 82**	2.29	3.87	0.43	0.16	**RF+ n = 82**	1.01	1.19	0.13	0.32
**RF-n = 40**	2.28	3.34	0.54		**RF- n = 40**	1.37	1.64	0.28	

There were no differences in miR-155 plasma expression and its gene methylation between various treatment options. The results are presented in the table and figure in S2 Table and S1 Fig.

## 4 Discussion

### 4.1 MiR-155 methylation and miR-155 plasma expression in RA vs HC

We describe here significant differences in miR-155 gene methylation levels between RA and HC; with significantly higher methylation levels in RA patients. These results are consistent with the general epigenetic principle that over-methylation of the promoter region of a gene leads to a decrease of its protein synthesis. As expected, we confirmed that high miR-155 gene methylation levels are associated with lower plasma expression of miR-155.

### 4.2 MiR-155 methylation in RA patients

Inside the RA study group, we noticed a tendency for higher methylation levels and lower miR-155 plasma expression in patients with DAS28 higher activity scores, but this was not statistically significant. There is little literature on miR-155 methylation levels in RA, but similar results were found in other conditions, especially cancers. In prostate cancer, for example, miR-155 promoter methylation was predictive of biochemical prostate cancer recurrence after radical prostatectomy and might be useful as a diagnostic or prognostic marker [22]. MiR-155 gene methylation and miR-155 expression are also associated with multiple myeloma outcome [23].

### 4.3 Plasma miR-155 concentration

We have confirmed significantly lower miR-155 plasma concentrations in RA vs HCs. There are contradictory reports concerning miR-155 levels in serum or plasma in RA. Some authors report higher levels of miR-155 in RA patients compared to controls, while others found lower levels [24–26]. In specific blood cells or synovial tissue (i.e. PBMCs, RAFSs) researchers agree that miR-155 expression is up-regulated in RA patients [27, 28]. According to Murata et al. plasma and synovial fluid miR-155 concentration have different profiles in the same patients. Synovial fluid miR-155 concentration was higher in RA vs HCs and there were no statistical differences in its plasma expression between RA and HC. The authors demonstrated a negative correlation between plasma miR-155 expression and tender joint number (TJN) in RA patients and positive synovial miR-155 correlation with TJN, without any correlations with DAS28 [18]. Our results are also contrary to the findings of Anaparti et al. They found over-expression of miR-155, miR-146a-5p, miR-103a-3p, miR-26b-3p in RA vs HC in whole blood [29]. Other authors discovered an increased miR-155 expression in PBMCs in RA patients and its positive correlation with CRP, DAS28, TNF-α and IL-1β [30, 31]. We found a negative correlation of plasma miR-155 concentration and DAS28, ESR and CRP. It is very difficult to compare miR-155 results in various compartments. Some authors suggest that there is discordant expression of miR-155 even between PBMC and whole blood cells while others demonstrate convergence between these two expression patterns [24, 32]. Similar discordance is found in other diseases. The expression of miR-10a-5p in patients with diabetes mellitus type 1 (T1D) was found down-regulated in pancreas tissue and up-regulated in PBMCs or serum. MiR-146 expression in the same disease was found elevated in T-cells and decreased in serum and PBMCs. A similar situation was described regarding miR-16p, miR-25-3p, miR-9-3p and others in T1D [33]. Another example is miR-150, which is overexpressed in plasma and serum exosomes and downregulated in dermal fibroblasts exosomes in Systemic Sclerosis [34].

The miR-155 results analysis in various studies show that results are discordant especially in the plasma compartment. It was confirmed that miRs exist in plasma in highly stable forms which might be precisely and repeatedly measured [35]. Micro-RNAs in plasma might be stabilized and transported in exosomes released from various cells. Exosomes transport miRs and proteins between distant areas of the body, spreading inflammation. miR stabilization may also occur through RNA-induced silencing complexes (RISC) [18]. The presence of various miRs in the extracellular space differ depending on the compartment (synovial fluid vs plasma). This is likely due to the fact that they originate from different cells. MiRs consumed in the nuclei and cytoplasm of the cells during intensive inflammation might be present in plasma in lower concentration, but when miRs are released into the circulation from injured tissues like kidney, heart or liver, their expression may increase [36]. This may explain the reduced plasma concentration of pro-inflammatory miR-155 during RA.

### 4.4 RA sero-positivity and miR-155 concentration

We have compared plasma miR-155 expression in ACPA and RF positive RA patients vs negative ones. We have not found differences in levels in the plasma compartment. Other authors have previously confirmed that monocytes have higher miR-155 expression in ACPA positive vs ACPA negative RA patients [31]. Similarly, it was found in peripheral blood derived CD19^+^ cells, there was a higher concentration of miR-155 in RA ACPA (+) patients than RA ACPA (-) or HC [15].

### 4.5 The role of miR-155 in rheumatoid arthritis

MiR-155 has many functions that affect RA pathogenic pathways. MiR-155 decreases SOCS1 (Suppressor of Cytokine Signaling 1) levels, leading to up-regulation of pro-inflammatory cytokines such as TNFα or IL-1β in PBMCs and is over-expressed in Th17 cells [30, 37]. MiR-155 expression is elevated in synovial fluid monocytes (SFM), RASF and PBMCs and might increase their resistance to spontaneous and Fas-mediated death resulting in monocytes/macrophage accumulation in the synovial tissue and overproduction of pro-inflammatory cytokines [4, 38–40]. The majority of these cells in the affected joints are recruited from the blood [41]. Recent studies indicate that the migration of peripheral blood monocytes (PBM) to the synovium is facilitated by miR-155 chemokine-dependent regulation. Expression of miR-155 in CD14^+^ PBM in RA patients is higher than in HC and miR155 is involved in overexpression of chemokines (CCL) CCL3, CCL4, CCL5, CCL8 and chemokine receptor (CCR) CCR2 and CCR7. These data support a possible mechanism through which miR-155 could stimulate inflammation in the joints via recruitment and retention of leucocytes from the blood to the synovium [40].

Aliverini et al. found miR-155 over expression in synovial and peripheral blood B cells, especially in the early phases of RA. They suggest that miR-155 has a significant impact on B cell activation, differentiation, antibody production and IgG class switching via miR-155/PU.1 transcription factor pathway. The second important miR-155 cell targets are macrophages. Elevated expression of miR-155 in these cells results in lowering SHIP-1 activity leading to overproduction of TNFα and other inflammatory cytokines [15, 42].

Plasma miRs are derived from various cells. Measurement of miR-155 expression in plasma and evaluation of its gene methylation in cells from the whole blood are feasible, but might be difficult to interpret. This study was not specifically designed to evaluate the origin of circulating miR-155, but to find out if it is possible to use its plasma expression as a marker of RA or RA activity. We found significantly lower concentrations in the plasma of RA patients vs HC.

We confirm here that in non-cancer conditions, miR-155 gene expression is regulated by its promoter methylation. This mechanism might be used in the future as a treatment target.

## 5 Conclusion and remarks

As was shown before there are significant differences between expression of various miRs between cells in various compartments in the same individual. Our research shows that even if miR-155 is over-expressed locally in PMNCs or RASFs in RA patients, its expression in plasma is decreased. It might be of great interest to evaluate miR-155 gene methylation level in various specific cell lines, to evaluate if there is consistency between blood cells sub-populations, RASFs and others. It may confirm its role in the initiation and maintenance of RA. The lowering of miR-155 plasma expression might be significant in evaluation of inflammation in RA, however it will require further study. Concerning miR-155 gene methylation, we confirmed that this gene undergoes epigenetic regulation via its promoter CpG islands methylation. We were unable to correlate levels of miR-155 plasma concentration or its gene methylation with different RA treatments due to our small sample size and the considerable diversity of treatments. Further investigations are needed to investigate these associations.

MiR-155 appears to be a pivotal mediator which activates innate and adaptive immunity in RA. The measuring of miR-155 expression in plasma and evaluating its gene methylation in cells from the whole blood are easy to obtain but might be difficult to interpret. We found higher miR-155 methylation level in whole blood derived DNA and lower plasma miR155 expression in RA in comparison to HC. The evaluation of miR-155 host gene methylation status or miR155 plasma level are potentially useful as a marker for RA, but further investigations are warranted.

## Supporting information

S1 Table The raw data from the study. (DOCX)

S2 Table MiR-155 methylation and expression levels according to the way of treatment. (XLSX)

S1 Fig MiR-155 expression and methylation in the treatment groups. (DOCX)
