# Supplementary material for: Hypermethylation of the miR-155 gene in the whole blood and decreased plasma level of miR-155 in rheumatoid arthritis
Source: PLoS One. 2020 Jun 2;15(6):e0233897. doi: 10.1371/journal.pone.0233897 (PMC7266293; doi:10.1371/journal.pone.0233897)
Supplement: S1 Table — (DOCX) [file pone.0233897.s001.docx]

# **SUPPLEMANTARY DATA.**

**Hypermethylation of the miR-155 gene in the whole blood and decreased plasma level of miR-155 in Rheumatoid Arthritis.**

**Kolarz B. et al.**

Table S1. Mir-155 methylation and expression levels in comparison to treatment.

| **Group** | **miR155 Methylation**  **[unmethylated sequences]** | | **miR155 Expression** | |
| --- | --- | --- | --- | --- |
|  | Mean Fold-change | STD Error | Mean Fold-change | STD Error |
| **MTX, n=22 (26.2%)** | 1.85 | 0.27 | 0.97 | 0.25 |
| **MTX+Steroids, n=30 (35.7%)** | 2.47 | 0.9 | 1.29 | 0.31 |
| **MTX+Biologics, n=15 (17.9%)** | 2.47 | 0.9 | 1.14 | 0.24 |
| **Steroids, n=17 (20.2%)** | 1.97 | 0.64 | 0.83 | 0.21 |
| **p-value between treatment groups** | 0.48 | | 0.31 | |
| **Overall**  **n=84** | 2.21 | 0.38 | 1.09 | 0.14 |

Abbreviations: MTX; methotrexate.

Figure S1. Mir-155 expression and methylation between treatment groups.


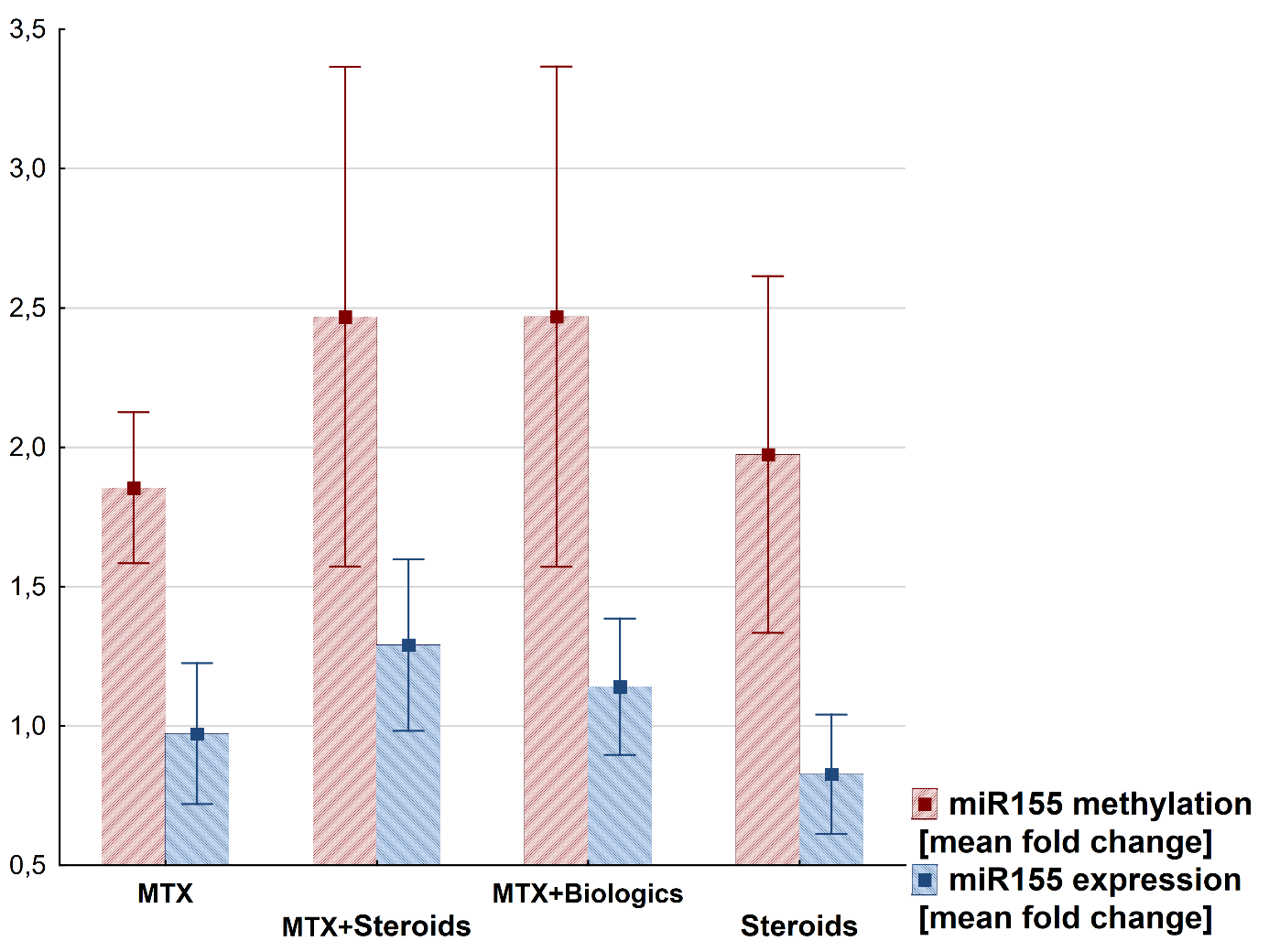


Data are given as: mean ± std error. Abbreviations: MTX; methotrexate.
