# Supplementary material for: Hypermethylation of the miR-155 gene in the whole blood and decreased plasma level of miR-155 in rheumatoid arthritis
Source: PLoS One. 2020 Jun 2;15(6):e0233897. doi: 10.1371/journal.pone.0233897 (PMC7266293; doi:10.1371/journal.pone.0233897)
Supplement: S1 Fig — (DOCX) [file pone.0233897.s003.docx]

**S1.** **Fig. Mir-155 expression and methylation between treatment groups.**

**Kolarz B. et al.**


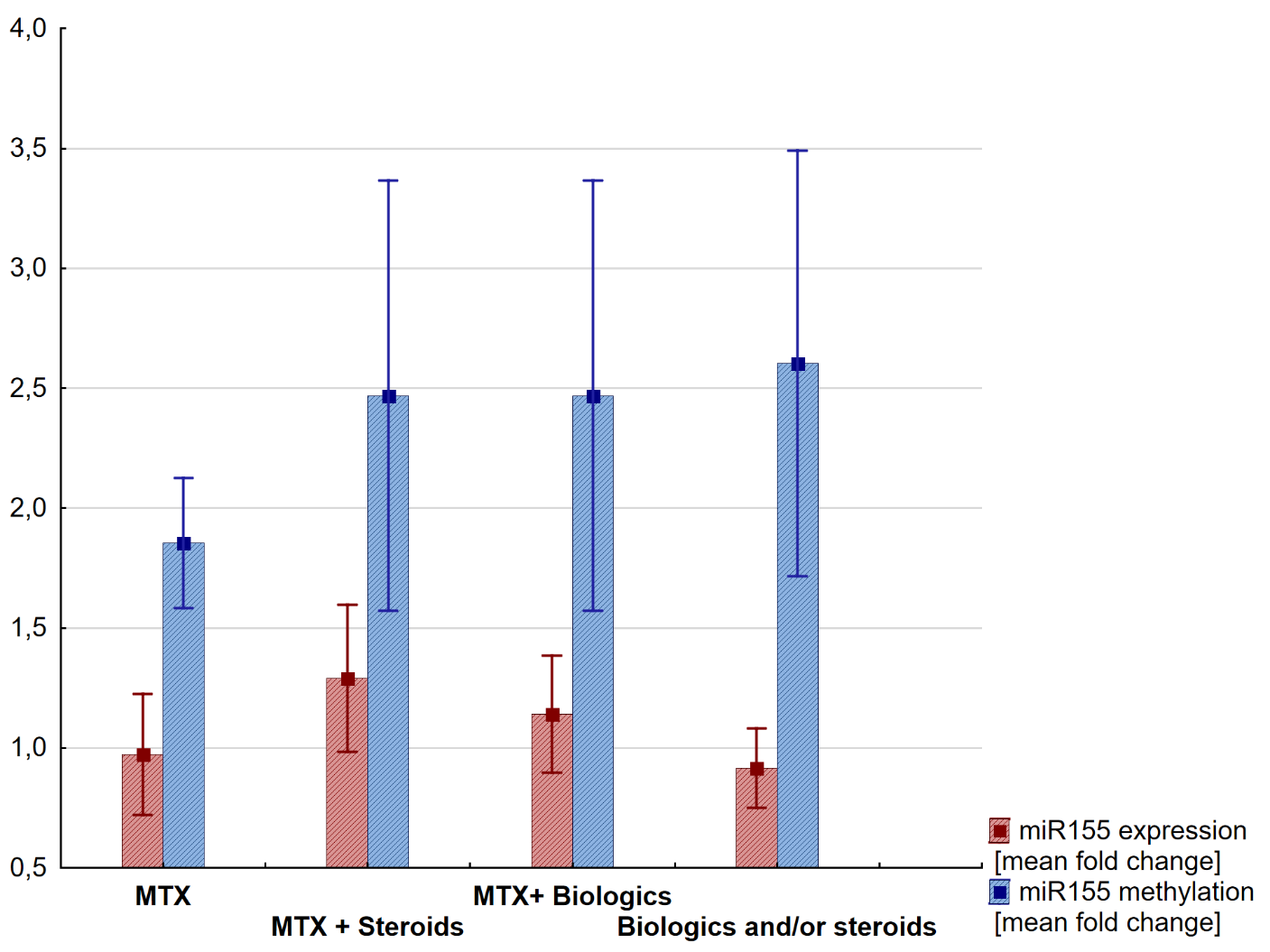


*Data are given as: mean ± std error. Abbreviations: MTX; methotrexate.*
